# Health and Welfare Survey of 30 Dairy Goat Farms in the Midwestern United States

**DOI:** 10.3390/ani11072007

**Published:** 2021-07-05

**Authors:** Melissa N. Hempstead, Taylor M. Lindquist, Jan K. Shearer, Leslie C. Shearer, Paul J. Plummer

**Affiliations:** Veterinary Diagnostic and Production Animal Medicine, College of Veterinary Medicine, Iowa State University, Ames, IA 50011, USA; melissa.hempstead@agresearch.co.nz (M.N.H.); tml@iastate.edu (T.M.L.); jks@iastate.edu (J.K.S.); lshearer@iastate.edu (L.C.S.)

**Keywords:** animal welfare, animal husbandry, welfare assessment, wellbeing, goat, caprine, dairy

## Abstract

**Simple Summary:**

There appears to be a rapid expansion of dairy goat farming in the United States and the information available to producers on health, welfare, and production applicable to those in the Midwestern US is limited. This study intended to survey 30 dairy goat farms in the Midwestern US to provide insight into husbandry practices pertaining to health, welfare, and production, and to identify areas of future research. Pain relief for disbudding and castration, education and training programs, early kid management, and hoof trimming were identified as potential areas of future research. This study provided insight into the husbandry practices carried out on 30 dairy goat farms in the Midwestern US and areas of research to improve health and welfare.

**Abstract:**

Dairy goat production in the Midwestern United States is increasing at a rapid rate and information on dairy goat husbandry practices applicable for producers in this region is limited. The objective of this study was to survey 30 dairy goat farms in the Midwestern US to provide insight into husbandry practices pertaining to health, welfare, and production, and to identify areas of future research. A questionnaire was developed and comprised 163 questions that were organized into categories including information on the producer (e.g., farming experience), staff, and goats (e.g., herd size, breed), housing, feeding and nutrition, milking practices and production, kid management, husbandry practices (e.g., disbudding, castration, hoof trimming), and health. Areas of future research that can improve goat health, production and welfare include pain relief for husbandry practices such as disbudding and castration, early kid management during birth to prevent illness/disease or mortality (e.g., warm and dry areas for kid rearing), eradication programs for common contagious diseases, training programs and education for claw trimming, disbudding, and udder health. In conclusion, this study provided insight into the husbandry practices carried out on 30 dairy goat farms in the Midwestern US and areas of research to improve health and welfare.

## 1. Introduction

In the United States (US) there are approximately 2.7 million goats, and of these, 440,000 are dairy goats [1]. The number of milking goats within the study population comprising Minnesota, Iowa, Wisconsin, and Illinois is 14,000, 29,000, 82,000 and 10,000, respectively [1], which is representative of approximately 30% of the total population of dairy goats in the US.

To date, information on dairy goat husbandry practices applicable for the Midwestern US is limited, which may be due to them historically being regarded as a minor species in comparison with dairy cattle [2]. However, demand for goat milk products, such as cheese and yoghurt, is rising and likely related to changes in demographic composition, which has driven the proliferation of dairy goat operations throughout the US [2].

Welfare assessment of dairy goat farms is increasing, with research available in the UK [3], Europe [4,5,6,7] and South America [8]. However, to our knowledge, there are few studies of dairy goat welfare assessment in the US, with the exception of our own study [9].

The objective of this study was to survey 30 dairy goat farms in the Midwestern US (enrolled in a welfare assessment study [9]) to provide insight into current husbandry practices pertaining to health, welfare, and production, and to identify potential areas of future research. Based on our knowledge of the Midwestern US dairy industry, we hypothesized that producers would have knowledge gaps in the areas of early kid management, and routine husbandry procedures, such as disbudding and hoof trimming.

## 2. Materials and Methods

This study was part of a larger study that performed welfare assessment of lactating dairy goats from 30 farms across the Midwestern US and identified the most prevalent welfare issues [9]. The present study was conducted between January and December 2019 and surveyed human participants; therefore, the survey protocol was reviewed by the Iowa State University Institutional Review Board (IRB; No. 18-497) prior to study commencement. Our protocol was deemed exempt from IRB approval. However, we ensured that the rights and privacy of the study participants were protected. After providing informed consent to be involved in this study, participants were given an identification number between 1 and 30, which was used to de-identify the collected data and distinguish between individual responses. The first author had access to the personal details of the participants, which were linked to their unique identification number on a computer that was stored in a lockable filing unit when not in use.

### 2.1. Survey Development

The questionnaire used to survey producers was developed from a review of available literature on existing surveys of goat producers, goat welfare assessment, producer attitude to goat behavior and welfare, husbandry practices and farm management [5,6,10,11,12,13,14] and the researchers’ own experiences with dairy goat farming. The questionnaire was designed using a web-based data collection software (REDCap^®^, Vanderbilt University, Nashville, TN, USA). Questions were entered into REDCap^®^, which generated the questionnaire.

The draft questionnaire was initially reviewed by a small committee of veterinary practitioners and an animal scientist, and a social scientist in the Department of Sociology of Iowa State University. The modified draft questionnaire was then reviewed by eight members of the American Dairy Goat Association, a representative of a milk company operating within the Midwestern US, and two commercial dairy goat producers. Based on these reviews, a final version of the questionnaire was produced. The questionnaire comprised 163 questions that were organized into 11 major topics including producer-specific information (e.g., farming experience), staffing, goat-specific information (e.g., herd size, breed), housing and environment, goat behavior, feeding and nutrition, milking practices and milk production, goat kid management, husbandry practices (e.g., disbudding, castration, hoof trimming, euthanasia), cleaning and sanitation, and health and veterinary care. Information on participant demographics were also collected. The questionnaire included a mixture of multiple choice, rating scale/slider, Likert scale, matrix, and open-ended questions. The questionnaire took approximately 60 min to complete based on responses from those that reviewed the questionnaire during the drafting phase.

A blank copy of the questionnaire is available in the Supplementary Materials (Table S1).

### 2.2. Study Participants

Producers were recruited by distribution of advertising material by a milk company operating in the Midwestern region on our behalf, and visitation of the first author with a feed representative to directly distribute the advertising material over a month-long period. Participation was incentivized by receipt of compensation for the producers’ involvement in this study. Those that indicated their interest in this study were provided an introductory letter describing the study objectives and additional information to help them decide whether to commit to enrolling in this study. Producers were enrolled in this study over a 6-month period and the questionnaire was distributed once informed consent was received. Participation was voluntarily and producers were self-selected. Further details of farmer recruitment are described by Hempstead et al. [9].

### 2.3. Survey Delivery

Questionnaires were sent to producers between February and November and responses were received between February and December 2019. Producers were sent a link to REDCap^®^ (via email), which presented the questionnaire and collected their responses (18 participants). However, not all producers had email capabilities or computer access; therefore, a hardcopy version of the online questionnaire was mailed to these producers (12 participants). When required, producers were sent a reminder until all questionnaires had been received.

### 2.4. Data Management and Statistical Analysis

Data were exported from REDCap^®^ as a comma-separated values file and used with Excel (Microsoft Corporation, Redmond, WA, USA). The data were presented as (1) percentages, with the actual number of farms in brackets and means with standard error, or (2) mean values with standard deviation (SD) or interquartile range (where appropriate). Due to the self-selected nature of the study participants, no formal hypothesis testing was conducted.

## 3. Results

### 3.1. Response/Completion Rate

All 30 producers completed the questionnaire, which included one respondent who completed the online questionnaire in part twice; these data were combined, and any differences in reported values were averaged. No duplicate questionnaires were entered. Eighteen questionnaires were completed online and 12 were completed on hardcopy. The number of days for producers to return the questionnaire ranged from 0 to 175 days, with a mean of 34 days (SD = 46).

### 3.2. Demographics

Producers had a mean age of 44 (SD = 11; range = 17–62 years old) and 64% (18/28) of producers were male (36% were female [10/28]; age not disclosed for two participants). The mean number of years of experience farming goats was 14 years (SD = 14; range = 3–52 years), and 73% (22/30) of producers had experience on a cow dairy; for these respondents, the mean number of years of experience was 19 years (SD = 10; range = 1–38 years).

### 3.3. Producer-Specific Information

Producers rated how they felt about animal welfare and whether it was a key priority for how their farm was run along a rating/slider scale (where numbers 0 through 100 were presented along a scale; 0 = strongly disagree, 50 = neither agree nor disagree, 100 = strongly agree). The responses of 29/30 producers (one participant did not respond) ranged from neither agree nor disagree to strongly agree (mean ± SD = 90 ± 11). In addition, producers were asked to rate the level of importance they placed on staff training along a rating/slider scale (where 0 = not at all important, 50 = important, 100 = very important). The responses of 26/30 producers (four participants did not respond) ranged from important to very important (mean ± SD = 77 ± 5). However, three of the producers rated training between not at all important and important (≤37 ± 5). Producers were asked what type of training was provided to staff; 13% (4/30) of producers responded they did not provide any training. The types of training provided by the remaining participants are presented in Table 1. Interestingly, producers that rated training as between not at all important and important reported they provided staff training. Those that provided no staff training did not provide an explanation.

### 3.4. Farm Characteristics

Based on producer responses to the questionnaire, the median number of lactating does was 166 (IQR = 70), which ranged between 2 and 6500 does (mean ± SD = 618 ± 1519). The median herd size (i.e., number of lactating and non-lactating does) was 185 (IQR = 175), which ranged from 55 and 9000 does (mean ± SD = 788 ± 1964). The mean lactation length was 314 days (SD = 66; range = 190–600 days), with a mean yearly production of 7 pounds/doe/day (SD = 2; range = 5–11 pounds/doe/day). Ten percent (3/30) of producers responded as being certified organic. Ninety three percent of producers (28/30) farmed Saanen and Alpine breeds. American Lamancha, Anglo-Nubian, Toggenburg, Oberhasli, Sable and Kiko were present on 60% (18/30), 37% (11/30), 33% (10/30), 20% (6/30), 7% (2/30) and 3% (1/30) of farms, respectively.

### 3.5. Goat Husbandry

#### 3.5.1. Resources

The most common diet included hay and grain/concentrate, which was approximately four-times more popular than fermented forage or other feeds (Table 2). Access to outdoor spaces were provided on 73% (22/30) of farms (Table 2), of which earthen and pasture surface types were most common compared to concrete and rock piles (Table 3).

#### 3.5.2. Milking Procedures

Machine milking was used on 93% (28/30) of farms. Hand milking was used on 7% (2/30) of farms. Udder preparation prior to milking was conducted on 90% (27/30) of farms; this included cleaning the teats of debris on 89% (24/27) of farms, sanitizing the teats on 44% (12/27) of farms and checking the fore milk on 30% (8/27) of farms. Ten percent (3/30) of producers reported that no udder preparation was carried out. Udder treatment following milking was carried out on 70% (21/30) of farms, and 100% (21/21) of these farms used a teat dip or spray and 23% (5/21) of these farms used teat conditioner; 30% (9/30) of producers reported they did not use any post-milking treatment.

Eighty percent (24/30) of producers said that they actively check for evidence of mastitis. Of these farms, 100% (24/24) assessed milk quality, 79% (19/24) assessed swelling, 75% (18/24) assessed heat and firmness to the touch and 58% assessed udder color (14/24).

#### 3.5.3. Goat Husbandry Practices

All producers reported that claw trimming was carried out on the goats. Responses to questions on corrective claw trimming practice including who performs claw trimming and sources of training, the tool used, frequency and average age of goat at first claw trim are presented in Table 3.

Producer responses relating to health including treatment for gastrointestinal and external parasites, pain relief usage, and veterinary experience with goats are presented in Table 4. Producers rated how often veterinary treatment was sought along a rating/slider scale (where 0 = never, 50 = sometimes, 100 = always). The responses of 27/30 producers (three participants did not respond) ranged from never to sometimes (mean ± SD = 49 ± 22).

Producer responses relating to diagnosis of diseases on their farms including caseous lymphadenitis (CL), caprine arthritis encephalitis (CAE) and Johne’s disease are presented in Table 5. Approximately two-thirds of producers reported having CL on their farms, with just under half of participants having diagnosed CAE; Johne’s disease was less commonly diagnosed on farms (Table 5).

### 3.6. Goat Kid Husbandry

Producer responses relating to goat kid rearing practices including removal from dam after birth (a practice common place in goat farming to reduce the risk of disease transmission such as CAE), rearing method, colostrum management, navel disinfection, housing strategies, and weaning age are presented in Table 6.

Producer responses relating to disbudding including method, operator and training received, goat kid age at disbudding, power source, iron application time, horn bud removal and antiseptic application are presented in Table 7. Producers rated how confident they felt in their practice to disbud kids effectively without complication along a rating/slider scale (where 0 = not confident at all, 50 = confident, 100 = very confident). The responses of all (26/26) of the producers that perform disbudding themselves ranged from confident to very confident (mean ± SD = 86 ± 16). Producers were also asked to rate the likelihood of changing their practice if there was a better option available (where 0 = not at all likely, 50 = likely, 100 = very likely). The responses of 25/26 producers (one participant did not answer) ranged from likely to very likely (mean ± SD = 63 ± 24). Although, 3/25 producers rated the likelihood of changing their practice between not at all likely and likely (≤24). Additionally, producers rated how much pain they think disbudding causes (where 0 = no pain, 50 = some pain, 100 = extreme pain). The responses of 24/26 producers (two participants did not answer) ranged from some pain to extreme pain (mean ± SD = 60 ± 16), but 3/25 producers rated the amount of pain caused as being between no pain and some pain (≤41).

Producer responses relating to castration including whether castration is performed and what buck kids are kept for, the method used, goat kid age at castration, the operator and training received are presented in Table 8. Producers rated how confident they felt in their practice to castrate kids effectively without complication along a rating/slider scale (where 0 = not confident at all, 50 = confident, 100 = very confident). The responses of 7/8 producers (one participant did not answer) that perform castration themselves ranged from confident to very confident (mean ± SD = 85 ± 18). Producers were also asked to rate the likelihood of changing their practice if there was a better option available (where 0 = not at all likely, 50 = likely, 100 = very likely). The responses of all (8/8) producers ranged from likely to very likely (mean ± SD = 62 ± 27). Although, 3/8 producers rated the likelihood of changing their practice between not at all likely and likely (≤32). Additionally, producers rated how much pain they think castration causes (where 0 = no pain, 50 = some pain, 100 = extreme pain). The responses of 6/8 producers (two participants did not answer) ranged from no pain to some pain (mean ± SD = 49 ± 23).

The key research areas that have be identified from this study are presented in Table 9.

## 4. Discussion

Our study has provided insight into the current husbandry practices of 30 dairy goat producers in the Midwestern US and highlighted potential areas of future research to improve dairy goat health, production, and welfare.

### 4.1. Producer-Specific Information

The producers involved in the present study largely agreed that good animal welfare was a key priority for their farms; however, whether this correlates with actual positive welfare on-farm is not yet understood [9]. Some research suggests that consumer preference to buy products from animals that experience a high level of animal welfare does not necessarily correlate with those products being purchased, and that consumers may select products based on price and not ethical standards [15].

Training of staff responsible for animal care and management is crucial for providing a high standard of animal health and welfare [16,17,18]. Handlers that received both hands-on and online training on cornual nerve block application for cautery disbudding of calves had a much higher success rate and better outcomes for the calves undergoing this procedure than those operators that received online training only [18]. A large-scale study of dairy cattle farms in southern and central Italy reported that levels of staff training were inversely related to prevalence failures in almost all areas of welfare assessed; for example, farms that had no parasite control, foot bathing, routine footcare, or vaccination programs also failed to provide animal welfare training for stock people [17]. Combined, this research demonstrates the importance of training specific for animal welfare in order to ensure good welfare outcomes for livestock. Most producers in this study rated highly the importance of staff training; however, a small number of those producers reported they did not provide training (13%). These results may suggest that some producers responded as they thought they should, rather than what they actually thought. Those that did not provide training were generally small family-run farms and responded that they worked with goats from a young age. A recent study from Turkey reported that 73% (67/92) of dairy goat producers did not provide staff training for milking practices, which may not have been necessary as the producers stated that staff had many years of milking experience [19]. Additionally, Gökdai, Sakarya, Contiero and Gottardo [19] reported that producers were aware that training was a necessity, but that time constraints prevented them from providing adequate training to their staff. Training programs specific to dairy goat producers in the Midwestern US setting are required and should be actively encouraged to ensure stockperson uptake.

### 4.2. Goat Husbandry

Access to outdoor spaces was provided on approximately three-quarters of the farms in this study, and over half provided the goats the opportunity to graze on pasture, with more than one-quarter of producers providing access to concrete or rocks; this may reflect producer preference for goats to have access to environments that encourage performance of natural behaviors, or contain enrichment (e.g., climbing structures, pasture). A survey of 46 dairy goat farms in the UK reported that 17% of farms grazed goats outdoors, which the authors postulated was associated with the difficulties maintaining a high-yielding herd on pasture and the susceptibility of goats to parasitism with gastrointestinal nematodes (GIN) [10]. Reducing parasite load was also cited as an explanation for 12/13 dairy goat farms in New Zealand maintaining their does in intensive production (i.e., housed in barns where forage and crops are transported and fed to goats) [20]. Differences in availability of outdoor spaces between the Midwestern US and other nations may be due to variance in climatic conditions that allow GIN to be problematic (e.g., decrease production). However, these explanations require validation.

Sixty-nine percent of producers reported treating their does for GIN at least once a year. The GIN eggs are passed in the feces, and onto the pasture where the larvae hatch and are ingested during grazing [21]. As stated earlier, many producers in this study provided the goats with access to pasture, justifying regular worming regimens. However, anthelmintic resistance in nematode populations is a major issue in goats [22]; resistance to anthelmintics is associated with multiple factors including the fast metabolism of goats of drugs leading to shorter residence time facilitating nematode resistance and over use of anthelmintics at incorrect dosage [23]. Targeted selective treatment of individuals with high fecal egg counts has been suggested as a useful method of preserving anthelmintic efficacy by ensuring a number of untreated GIN in refugia [24]. Only two producers from the present study responded as using this technique. Another strategy involves the use of tannin-rich plants, which have natural anthelmintic properties to control nematode populations in goats [25]. Future research on tannin-rich plants that consistently show efficacy in vitro or the supplementation of grazing sheep with products containing Duddingtonia fungi to reduce pasture larvae [26] as well as better education programs for producers on careful GIN management to reduce resistance to anthelmintic drugs are required.

More than one-quarter of producers provided concrete in the outdoor area. Hard surfaces such as concrete may increase natural wear of the hooves. Additionally, goats spent more time lying on rubber matting and plastic slats than wood shavings indicating that goats may prefer solid surfaces to lie on than straw or wood chip [27]. Whether concrete and other solid surfaces are preferable over soft bedding materials and natural hoof wear is observed remains to be seen.

Ninety percent of producers reported that udder preparation was carried out prior to milking with physical removal of debris on the teats being most common. However, less than half of the farms sanitized the teats. A survey of 46 dairy goat farms in the UK, reported that 83% of farms used some form of udder and teat preparation [10]. Teat sanitizing dairy cattle prior to milking reduces the presence of *Staphylococcus aureus* (common mastitis pathogen affecting cattle) in milk [28]. The difference in these practices may be associated with the difference in acceptance of somatic cell count (SCC) levels by milk companies (1.5 million somatic cells and 750,000 somatic cells for goats and cows, respectively); therefore, cow dairies must reduce bacteria as much as possible. Multiple factors are associated with increased SCC in goats (compared with dairy cattle), such as increased dry matter intake, lactation number/parity, stage of lactation, and lower mature equivalent milk production [29]. Further, increased SCC is not necessarily correlated with intra-mammary infections [29]. Results of the National Animal Health Monitoring Survey in 2009 reported that 2.8% of lactating does in the US had clinical mastitis and 30.7% of operations had at least one doe with clinical mastitis; the most common form of identification was visual inspection [30]. Interestingly, 20% of producers we surveyed did not check for mastitis, indicating that the previously reported prevalence rate may be underrepresented. More education and training surrounding udder health for dairy goats in the Midwestern US is required.

Corrective claw trimming to remove excess claw growth is an important husbandry practice for goats’ claws that are not worn naturally. Severely overgrown claws (and diseases such as laminitis and CAE) may be associated with lameness, which is a major health issue in dairy goats [3,31,32]. Claw trimming was conducted on all farms and most commonly by the producer. Although 51.4% of goats observed on these farms (2325/4520) had overgrown claws [9]. A recent survey of goat producers in the UK reported that all producers perform claw trimming [10] yet an earlier study from the same group identified claw overgrowth as a major issue [3]. In the present study, claws were trimmed most commonly every 3–4 months (43%) and 5–6 months (33%), which is similar to previous surveys of dairy goat farmer in the UK [10] (36% and 33% for every 3–4 months and 5–6 months, respectively). There was, however, a higher percentage of producers that trimmed every 1–2 months in UK [10] compared with the Midwestern US (16% vs. 7%). Together, these results may indicate that more frequent hoof trimming is required to prevent claw overgrowth. Future research should evaluate best practice recommendations for claw trimming frequency to ensure minimal rates of claw overgrowth.

Pain relief was used on 70% of farms in the present study with the most common reasons including injury and disease. Factors that affected the use of pain relief on farms were the associated costs (including veterinary personnel), and time taken to administer, but most producers used pain relief because of the benefits to the animal. The relatively high number of producers that used pain relief on their farms may be associated with the self-selected nature of this study in that producers who were interested in improving animal welfare opted into this study.

Producers reported that they felt the veterinary practitioner they used had adequate goat experience on 73% of farms. In comparison, 83% of 46 producers in the UK felt their local veterinary practitioner had sufficient knowledge and experience with dairy goats [10]. Recent data from a USDA survey suggests that the number of goat producers (including meat, fiber and dairy) in the US consulting with veterinarians has increased, rising from 39.5% in 2009 to 49.7% in 2019 [33].

More than half of the producers reported that CL was diagnosed on their farms, with just under half reporting diagnosis of CAE on their farms. We are unaware of whether the producers in the present study were part of a testing or eradication program or the financial implications associated with these diseases, but this information would be useful to include in future studies. In the UK, producers observed these diseases far less often (CL: 7%, 10/45 farms; CAE: 11%, 5/45 farms) [10]. The relatively high number of goats with these diseases on farms in the present study may be associated with non-selective breeding for animals that are susceptible to these conditions, or management practices that increase the risk of transmission such as a failure to separate or cull infected animals. Johne’s disease was the most commonly reported disease on UK dairy goat farms (49%; 22/45), whereas producers in the present study responded as having this disease on their farms less often (7%; 2/30 farms); this may be due farmers may not be actively checking for Johne’s disease. With the apparent trend of dairy goat farming expansion in the US, sourcing goats from multiple herds and comingling, likely increases the risk of disease transmission. Therefore, careful checking or testing of new animals brought into the herd prior to comingling, can reduce the risk of disease. A disease eradication program to control CAE, CL and Johne’s diseases may be beneficial for the Midwestern US dairy goat industry as has been largely successful on Norwegian dairy goat farms [34].

### 4.3. Goat Kid Husbandry

Interestingly, only half of the producers used heating in the kid rearing areas, even though the study area ambient air temperatures can reach −20 °C (or below) [35] in the winter during kidding season. In our recent study, approximately 65% (17/30) of farms had goats with frostbitten ears (i.e., any amount of pinna is missing; appears straight cut), which was likely caused by extended time spent in extreme low temperatures at birth [9]. It is vital that producers ensure better management of neonatal kids during extreme temperatures by completely drying kids after birth and moving them to warm environments. Goat kids that experience cold air temperatures (−3 to 10 °C) for at least 5 days after parturition have lower survival rates compared to kids exposed to warmer temperatures [36,37]. However, an earlier study observed no mortalities in kids raised in non-insulated rooms (−10 to −4 °C) compared to insulated rooms (9 to 14 °C) [38]. Adult goat behavior is affected by temperature as lying time was reduced in goats experiencing low (−6 to −8 °C) ambient air temperatures compared with moderate (10 to 12 °C) ambient temperatures [39]. Additionally, goats will spend reduced time in outdoor spaces (compared with indoors) with decreasing temperatures (below −10 °C) [40]. More education and training programs are required to increase the use of insulated or heated rooms for newborn kids.

The overwhelming majority of producers in this study used the cautery method to disbud goat kids (only one producer did not state the method used). Cautery disbudding (with the provision of pain relief) appears to be the best option to date for having hornless goats [41,42,43,44]. In the present study, 29% (6/21) of producers used pain relief for disbudding, which appears a relatively low number when compared to the UK of which all kids require anesthesia and/or analgesia for disbudding [45]. To improve the welfare of goat kids undergoing disbudding, it is important that some form of pain relief is used. Caustic paste disbudding is the second most common method used in calves [14,46,47], but it can run into the eyes or be rubbed onto other areas of the body or pen mates [48]. Additionally, caustic paste causes more pain in goat kids than cautery disbudding and may not consistently prevent scurs [41,42]. In a previous survey of goat producers and veterinarians in the US and Canada, 97% (39/40) of veterinarians and 95% (218/229) of producers stated they used a cautery iron to disbud their goat kids [49]. The authors stated that caustic paste was not used, but provided no insight into what method was used instead [49].

Disbudding was predominantly carried out by producers in the present study, which was likely to reduce costs associated with veterinary practitioners; only a small number of producers employed veterinary practitioners to perform disbudding. In comparison with the UK, only veterinary practitioners are permitted to disbud goat kids (with a cautery iron and under anesthesia) under UK law [45,50]. Additionally, just over one-fifth of the producers that disbudded kids themselves, were trained by a veterinary practitioner, which is a smaller percentage than those that were trained by family and friends (12/26; 46%), again likely due to reduced costs. Producers received cautery disbudding training for calves in Canada and Czech Republic in 98% and 85% of producers surveyed, respectively [14,51]; however, we found that only 60% of producers received any cautery disbudding training, highlighting the need for a standardized cautery disbudding protocol for goat kids and training programs for producers in the Midwestern US.

It is typically considered good practice to disbud goat kids once the horn buds are palpable and within a week of age [52]. In a recent review of comparisons of disbudding practices between calves and kids, on average kids were disbudded at 10.6 days of age (SD = 5.7) (on average calves were 5.3 weeks of age at disbudding (SD = 2.0)) [53]. The majority of producers (77%) in the present study disbudded kids less than 2 weeks of age, although 23% of producers in the present study disbudded kids at an advanced age (i.e., more than 2 weeks of age). In the UK, kids are commonly disbudded at less than 2 weeks of age (93%; 42/45) [10]. Disbudding goat kids beyond 2 weeks of age increases the difficulty of completely removing the horn bud and may increase the incidence of scurs and prolong healing [53], further highlighting the need for producer education and training in this area.

Seventy percent of producers used the cautery iron for 8 s or more to disbud their kids, which may increase the risk of thermal injury to the brain. In a recent pilot study by our group on the effect of cautery iron application duration on brain injury in goat kids (by a trained and experienced operator), we observed brain injury in at least some goat kids at all duration applications (5, 10, 15, and 20 s); however, longer applications resulted in more severe and consistent brain injury [54]. Application times of 15 and 20 s should be avoided as these durations resulted in severe histopathological changes, including a branching region of edema across multiple gyri, hemorrhage, and microscopic lesions consisting of leptomeningeal and cerebrocortical necrosis [54]. Cautery disbudding training should be included as part of routine training programs for farm staff by a veterinary practitioner or an experienced operator.

In the present study, 27% of producers performed castration on their animals, and of these producers, most buck kids were castrated for reducing odor in the meat or because they were pets. The producers that did not perform castration tended to keep bucks for breeding purposes. Castration was carried out almost exclusively by the producer and with a ring or band, most likely as the practice requires minimal training and is not as technical as surgical castration. Ring castration does, however, cause acute and long-term pain in lambs (reviewed by [55,56]). Use of the Burdizzo method appears to cause similar behavioral and stress responses as ring castration in lambs, which can be reduced by local anesthesia and non-steroidal anti-inflammatory drugs [57]. Producers rated themselves as confident to very confident they could castrate their kids effectively without complication, although they were open to changing their practice if there was a better method available. This may be associated with using another method, which may be more time efficient. Producers rated castration as causing no pain to some pain. Future studies should investigate pain or distress associated with ring castration of goat kids.

This study was not without limitations. Ideally, we would have selected 30 farms at random from the Midwestern region to be involved in this study, but we were unable to access a database of producers in this region. Therefore, we must be cautious when trying to extrapolate our findings and make statements about the wider Midwestern dairy goat industry as there is likely to be some degree of self-selection bias, with consequent overestimation (or under-representation) of certain themes. For example, farmers that chose to be involved with this study likely already had an interest in improving animal welfare, therefore we may not have captured the views of those that had no interest in improving the welfare of their goats. In future, the survey should be made available to all producers within the Midwestern US region as a standalone survey without requiring a farm visit to perform welfare assessment. Further, it would have been useful to include a greater number range within the rating/slider scale to increase the sensitivity about the data collected.

## 5. Conclusions

In conclusion, this study has provided insight into the husbandry practices carried out on 30 dairy goat farms in the Midwestern US, which can be used by the industry to inform and improve routine husbandry practices.

Our study has highlighted many potential areas of future research to improve the welfare of dairy goats. Major potential areas that showed the highest number of producers that did not perform various husbandry procedures were the use of pain relief for husbandry practices such as castration and disbudding, early kid management during birth to prevent illness/disease or mortality (e.g., warm and dry areas for kid rearing), eradication programs for common contagious diseases such as CAE and CL, training programs and education for claw trimming, disbudding, and udder health. Minor research areas included the highest number of producers that performed various husbandry practices such as the effect of outdoor space on goat behavior, hoof wear, parasite burden and productivity, corrective claw trimming frequency and the effect on claw growth, and parasite management.

## Figures and Tables

**Table 1:** Type of staff training provided by dairy goat producers.

Staff Training	Percentage (Number) of Farms
Animal handling	81% (21/26)
Goat behavior	46% (12/26)
Kid rearing practices	88% (23/26)
Identifying sick/injured animals	85% (22/26)
Feeding/nutrition	65% (17/26)
Routine husbandry procedures (e.g., ear tagging, disbudding)	77% (20/26)
Machinery/equipment operation	50% (13/26)
Milking routines	92% (24/26)
Housing	38% (10/26)
Transportation of goats	27% (7/26)
Record keeping	62% (16/26)
Other—genetics and breeding	4% (1/26)

**Table 2:** Dairy goat producer responses relating to resource availability.

Resources	Category	Percentage (Number) of Farms
Diet	Hay	90% (27/30)
	Grain/concentrate	90% (27/30)
	Fermented forage	23% (7/30)
	Total mixed ration (fermented and fresh forage)	10% (3/30)
	Fresh cut grass	7% (2/30)
	Corn	3% (1/30)
	Other—banana and watermelon peel	3% (1/30)
Outdoor pen surface	Earthen	86% (19/22)
	Pasture	59% (13/22)
	Concrete	27% (6/22)
	Rock piles	9% (2/22)

**Table 3:** Dairy goat producer responses relating to claw trimming practices.

Claw Trimming Practice	Category	Percentage (Number) of Farms
Operator	Producer	87% (26/30)
	Staff	20% (6/30)
	Friends or family	40% (12/30)
	Paid contractor(s)	27% (8/30)
Producer training	None, self-taught	42% (11/26)
	Trained by friends or family	46% (12/26)
	Trained by a veterinarian	8% (2/26)
	Trained by a paid contractor	4% (1/26)
	Other—reading material	8% (2/26)
Tool used	Grinder	10% (3/30)
	Hand-powered trimmer or shears	83% (25/30)
	Pneumatic hoof trimmer	13% (4/30)
	Blade	7% (2/30)
Frequency	Every 1–2 months	7% (2/30)
	Every 3–4 months	43% (13/30)
	Every 5–6 months	33% (10/30)
	Every 7–12 months	10% (3/30)
	When needed	7% (2/30)
Goat age at first claw trim	<3 months	3% (1/30)
	3–5 months	10% (3/30)
	6–8 months	33% (10/30)
	9–12 months	40% (12/30)
	Over 12 months	13% (4/30)

**Table 4:** Dairy goat producer responses relating to routine health practices.

Health	Category	Percentage (Number) of Farms
Frequency of treatment for gastrointestinal parasites	Every 3–5 months	14% (4/29)
	Every 6–8 months	17% (5/29)
	Every 9–12 months	38% (11/29)
	When needed	21% (6/29)
	Not clarified	10% (3/29)
Frequency of treatment for external parasites	Every 3–5 months	17% (5/29)
	Every 6–8 months	7% (2/29)
	Every 9–12 months	28% (8/29)
	When needed	21% (6/29)
	No treatment	7% (2/29)
	Not clarified	21% (6/29)
Pain relief usage	Yes	70% (21/30)
	No	30% (9/30)
Pain relief used for	Disbudding	29% (6/21)
	Castration	5% (1/21)
	Disease	67% (14/21)
	Injury	100% (21/21)
	Other—kidding	5% (1/21)
Factors that affect use of pain relief	Cost	10% (2/21)
	Time taken to administer pain relief	14% (3/21)
	Use of a veterinarian	5% (1/21)
	Benefits for the animal	90% (19/21)
	Benefits for humans (e.g., ease of handling)	10% (2/21)
Veterinarian used has adequate goat experience	Yes	73% (22/30)
	No	27% (8/30)

**Table 5:** Dairy goat producer responses to the question: has caseous lymphadenitis, caprine arthritis encephalitis or Johne’s disease ever been diagnosed on your farm?

Disease	Yes	No
Caseous lymphadenitis	64% (18/28)	36% (10/28)
Caprine arthritis encephalitis	48% (14/29)	52% (15/29)
Johne’s disease	7% (2/30)	93% (28/30)

**Table 6:** Dairy goat producer responses relating to common kid rearing practices.

Goat Kid Rearing Practices	Category	Percentage (Number) of Farms
Rearing method	Hand-rearing	97% (29/30)
	Dam-rearing	3% (1/30)
Removal from dam after birth	Immediately	76% (22/29)
	≤4 h of birth	17% (5/29)
	5–12 h	3% (1/29)
	at least 48 h	3% (1/29)
Amount of colostrum in 24 h after birth	≤10 ounces	27% (8/30)
	11–20 ounces	50% (15/30)
	21–30 ounces	3% (1/30)
	≥31 ounces	10% (3/30)
	Free choice	10% (3/30)
Colostrum type	Heat-treated goat colostrum	43% (13/30)
	Raw goat colostrum	20% (6/30)
	Powdered goat colostrum	33% (10/30)
	Powered cow colostrum	20% (6/30)
Navel disinfection	Yes	60% (18/30)
	No	40% (12/30)
Kid housing strategies (during winter months)	Electric/gas heating	50% (15/30)
	Heat lamps	33% (10/30)
	Insulated walls	40% (12/30)
	High pen walls to prevent drafts	37% (11/30)
	Hutches	7% (2/30)
Weaning age	5–6 weeks	10% (3/30)
	7–8 weeks	63% (19/30)
	9–10 weeks	23% (7/30)
	11–12 weeks	3% (1/30)

**Table 7:** Dairy goat producer responses relating to disbudding practices.

Disbudding Practices	Category	Percentage (Number) of Farms
Method	Cautery iron	97% (29/30)
	Not clarified	3% (1/30)
Operator	Producer	87% (26/30)
	Staff	13% (4/30)
	Friends or family	27% (8/30)
	Veterinarian	7% (2/30)
Training	None, self-taught	39% (10/26)
	Friends/family	46% (12/26)
	Veterinarian	23% (6/26)
Goat kid age at disbudding	≤7 days	33% (10/30)
	8–14 days	43% (13/30)
	15–21 days	13% (4/30)
	22–28 days	3% (1/30)
	≥29 days	7% (2/30)
Iron power source	Electric	66% (19/29)
	Liquefied petroleum gas (LPG)	10% (3/29)
	Gas canister (butane)	31% (9/29)
Total iron application time (per horn bud)	≤4 s	7% (2/29)
	5–7 s	21% (6/29)
	8–12 s	55% (16/29)
	≥13 s	17% (5/29)
Horn bud removal	Yes	62% (18/29)
	No	38% (11/29)
Antiseptic use	Yes	20% (6/30)
	No	80% (24/30)

**Table 8:** Dairy goat producer responses relating to castration practices.

Castration Practice	Category	Percentage (Number) of Farms
Castration performed	Yes	27% (8/30)
	No	73% (22/30)
Buck kids kept for	Showing	13% (1/8)
	Meat	88% (7/8)
	Pets	25% (2/8)
	Other—supplying an antigen laboratory	13% (1/8)
Method	Rubber ring/band	100% (8/8)
Goat kid age at castration	<1 week	13% (1/8)
	1–3 weeks	25% (2/8)
	4–6 weeks	13% (1/8)
	7–9 weeks	13% (1/8)
	10–12 weeks	38% (3/8)
Operator	Producer	100% (8/8)
	Staff	13% (1/8)
	Friends/family	13% (1/8)
Training received	No one, self-taught	38% (3/8)
	Friends/family	50% (4/8)
	Veterinarian	13% (1/8)

**Table 9:** Summary of the key areas of research for the Midwestern US dairy goat industry. The summary is arranged based on the percentage of producers (highest to lowest) that responded as *not performing* (major) or *performing* (minor) various husbandry practices.

Major Research Areas	Percentage (Number) of Farms
Effective and practical pain relief for castration	75% (6/8) thought castration did not cause pain
Effective and practical pain relief for disbudding	71% (15/21) did not provide pain relief for disbudding
Early kid management training and education (e.g., exposure to cold temperatures)	50% (15/30) did not use heating in kid rearing areas
Eradication or testing programs for common diseases (e.g., caseous lymphadenitis (CL), caprine arthritis encephalitis (CAE))	64% (18/28) and 48% (14/29) diagnosed CL, and CAE, respectively
Claw trimming training and education	40% (11/26) did not receive training
Standardized disbudding training and education	39% (10/26) did not receive training
Udder health	20% (6/30) did not monitor mastitis
General training programs for Midwestern US dairy goat producers	13% (4/30) did not provide staff training
Minor research areas	
Effect of outdoor space on goat behavior, hoof wear, parasite burden and productivity	73% (22/30) provided outdoor space
Corrective claw trimming frequency and claw growth	43% (13/30) trimmed claws every 3–4 months
Parasite management training and education	38% (11/29) drenched every 9–12 months

## Data Availability

The data presented in this study are available on request from the corresponding author. The data are not publicly available due to restrictions on privacy.

## Supplementary Materials

The following are available online at https://www.mdpi.com/article/10.3390/ani11072007/s1, Table S1: A blank copy of the questionnaire provided to dairy goat producers in the dairy goat welfare study.
