# Supplementary material for: Health and Welfare Survey of 30 Dairy Goat Farms in the Midwestern United States
Source: Animals (Basel). 2021 Jul 5;11(7):2007. doi: 10.3390/ani11072007 (PMC8300403; doi:10.3390/ani11072007)
Supplement: Supplementary file 1 [file animals-11-02007-s001.zip › animals-1238068-supplementary.pdf]

Dairy Goat Wellbeing Survey

Farm ID ______________________

Farm Owner

1 Why do you own a dairy goat farm?
- ☐ Continuing operation started by family member(s)
- ☐ I enjoy working with dairy goats
- ☐ Goats are smaller and easier to handle than cows
- ☐ My farm is small (i.e. space limitations)
- ☐ Good market for goat milk
- ☐ Other

Other - specify: ______________________

2 How do you feel about the following statement: The wellbeing of my goats is a key priority for how I run my farm.

Strongly disagree — Neither agree nor disagree — Strongly agree

(Place a mark on the scale above)

3. How important to you are each of the following wellbeing principles? Use a tick or cross to mark your answer.

	Not at all important	Slightly important	Important	Fairly important	Very important
Good feeding	◯	◯	◯	◯	◯
Good housing	◯	◯	◯	◯	◯
Good health	◯	◯	◯	◯	◯
Appropriate animal behavior	◯	◯	◯	◯	◯

4 How many years have you farmed dairy goats? ______________________

5 Do you have experience on a dairy cow farm? ◯ Yes ◯ No
(If yes, proceed to Question 6. If no, proceed to Question 7.)

6 How many years of experience? ______________________

7 Is your farm certified organic? ◯ Yes ◯ No

8 How often do you spend time with your goats (not including milking or routine procedures)?
- ◯ I do not spend time with my goats except for routine procedures, milking etc.
- ◯ Daily
- ◯ Weekly
- ◯ Monthly
- ◯ Other

Other - specify: __________________________________

9 Approximately how much time do you spend regularly watching your goats? __________________________________

10 Do you name your goats?
- ◯ Yes
- ◯ No

11 What data on your animals do you collect?
- ☐ I do not collect data
- ☐ Body weight
- ☐ Milk production
- ☐ Kid birth records (e.g., DOB)
- ☐ Death/mortality rates
- ☐ Sire/dam records
- ☐ Health
- ☐ Disease
- ☐ Genetic traits (e.g., without horn buds or polled)
- ☐ Other

Other - specify: __________________________________

12 Where do you go for information about operating a dairy goat farm?
- ☐ I do not seek information
- ☐ Veterinarian
- ☐ Other farmers
- ☐ Neighbors
- ☐ County extension service
- ☐ Dairy cooperative
- ☐ Family and friends
- ☐ Websites
- ☐ Social media
- ☐ State or national associations
- ☐ Online videos
- ☐ Magazines/newsletters
- ☐ Conventions/meetings
- ☐ Other

Other - specify: __________________________________

Staff (including paid or unpaid workers, family members)

13 How many staff (0-100) work on your farm (excluding you)? __________________________________

14 Are all of your staff responsible for handling the goats?
◯ Yes ◯ No
(If yes, proceed to Question 16. If no, proceed to Question 15.)

15 How many of your staff handle the goats on a daily basis? __________________________________

16 Is there a Code of Conduct for your farm?

◯ Yes ◯ No
(If yes, proceed to Question 17. If no, proceed to Question 18.)

17 Are your staff required to sign a contract confirming their compliance with the Code of Conduct?

◯ Yes ◯ No

18 How important to you is staff training?

Not at all important — Important — Very important

(Place a mark on the scale above)

19 What type of training does your staff receive?

- ☐ My staff do not receive training
- ☐ Animal handling
- ☐ Goat behavior
- ☐ Kid rearing practices
- ☐ Identifying sick/injured animals
- ☐ Feeding/nutrition
- ☐ Routine husbandry procedures (e.g., ear tagging, tattooing, disbudding, castration)
- ☐ Machinery/equipment operation
- ☐ Milking routines
- ☐ Housing
- ☐ Transportation of goats
- ☐ Record keeping
- ☐ Other

Other - specify: __________________________________

20 What factors affect your decision not to provide training for your staff?

- ☐ Training takes too much time
- ☐ Staff do not attend or engage
- ☐ It makes no difference for the staff or the animals
- ☐ I do not know what training is required
- ☐ I do not know where to go for training advice
- ☐ Other

Other - specify: __________________________________

21 Are documents signed by the employee(s) to confirm that training has been completed?

◯ Yes ◯ No

22 What type of training would benefit your staff?

- ☐ My staff would not benefit from training
- ☐ Animal handling
- ☐ Goat behavior
- ☐ Kid rearing practices
- ☐ Identifying sick/injured animals
- ☐ Feeding/nutrition
- ☐ Routine husbandry procedures (e.g., ear tagging, tattooing, disbudding, castration)
- ☐ Machinery/equipment operation
- ☐ Milking routines
- ☐ Housing
- ☐ Transportation of goats
- ☐ Record keeping
- ☐ Other

Other - specify: __________________________________

23 How would you prefer to receive training materials?
- ☐ Hard copy handout
- ☐ PDF on a device
- ☐ Via email
- ☐ Internet/website
- ☐ Magazine/newsletter
- ☐ Newspaper
- ☐ In-person training course
- ☐ Online training course
- ☐ At a group meeting/convention
- ☐ Other

Other - specify: __________________________________

Goats and Housing

24 How many does (adult females) are there in total on your farm? ____________________________

25 What breeds are being milked on your farm? If cross-bred goats, list the dominant breed(s).
- ☐ Saanen
- ☐ Toggenburg
- ☐ LaMancha
- ☐ Alpine
- ☐ Nubian
- ☐ Nigerian Dwarf
- ☐ Oberhasli
- ☐ Other

Other - specify: __________________________________

26 How many does are lactating? ____________________________

27 How many does are non-lactating (dry)? ____________________________

28 How many does will be (or have been) retired this year? __________________________________

29 How many doe kids will you keep (or have been kept) this year to replace your retired does? __________________________________

30 Do you have any goats used for purposes other than for milking (e.g., bucks, show goats)?
◯ Yes ◯ No
(If yes, proceed to Question 31. If no, proceed to Question 32.)

31 For what other purposes are your goats used?

☐ Bucks for breeding
☐ Show
☐ Meat
☐ Weed control
☐ Pets
☐ Other

Other - specify: __________________________________

32 Since January 1 2019, how many adult goats have died of natural causes? __________________________________

33 Since January 1 2019, how many adult goats have been euthanized? __________________________________

34 How do you group the goats into pens?

☐ Randomly grouped
☐ Age
☐ Weight/size
☐ Stage of lactation
☐ Stage of pregnancy
☐ Other

Other - specify: __________________________________

35 How often are the goats regrouped with goats from other pens?

☐ They are not regrouped (they stay in the same group)
☐ They are regrouped after each milking
☐ They may mix accidentally
☐ Other

Other - specify: __________________________________

36 How often is fresh bedding added to the pens? __________________________________

37 How often is the bedding in the pens cleaned out and replaced? __________________________________

Goat Behavior

38 How often do you think about the behavior of your goats?

Never — Sometimes — Always

(Place a mark on the scale above)

39 Please list all of the goat behaviors that you observe on a regular basis on your farm?

- ☐ I do not know
- ☐ Lying/standing in contact with other goats
- ☐ Feeding at the same time as other goats
- ☐ Drinking at the same time as other goats
- ☐ Running
- ☐ Walking
- ☐ Trying to escape the pens
- ☐ Kneeling for long periods
- ☐ Minimal vocalizing
- ☐ Vocalizing
- ☐ Rubbing their heads along the walls of the pen
- ☐ Standing facing pen walls for long periods
- ☐ Star gazing (looking skyward) for long periods
- ☐ Hair pulling of other goats
- ☐ Biting other goats
- ☐ Head butting
- ☐ Fighting
- ☐ Sneezing
- ☐ Other

Other - specify: ______________________

40 What allowances have you made on your farm for goats to perform natural behaviors?

- ☐ Hay racks
- ☐ Feed bunks
- ☐ Elevated food sources
- ☐ Shelter
- ☐ Places to hide
- ☐ Constant groups (i.e. no regrouping)
- ☐ Access to outdoor spaces
- ☐ Access to pasture/paddocks
- ☐ Enrichment (e.g., raised platforms, brushes)
- ☐ Adequate space per goat
- ☐ Other

Other - specify: ______________________

41 What do you think your goats are motivated to do (e.g., play, sleep, eat, move to parlor)? ______________________

Outdoor Access

42 Do your goats have outdoor access (not including pasture access)?

◯ Yes ◯ No
(If yes, proceed to Question 43. If no, proceed to Question 45.)

43 What type of surface(s) is available in the outdoor space?

☐ Earthen
☐ Concrete
☐ Gravel
☐ Wire mesh/metal
☐ Wooden slats
☐ Rubber matting
☐ Other

Other - specify:

____

44 Approximately how much time are they allowed in the outdoor space?

____

Feeding and Nutrition

45 Do your goats have access to pasture?

◯ Yes ◯ No
(If yes, proceed to Question 46. If no, proceed to Question 49.)

46 Approximately how much time do your goats spend on pasture?

____

47 Do your goats receive additional feed?

◯ Yes ◯ No
(If yes, proceed to Question 48. If no, proceed to Question 53.)

48 Where are the goats fed?

☐ Indoors
☐ Outdoors

49 What are the goats fed?

☐ Fresh cut grass
☐ Total mixed ration (both hay and silage)
☐ Silage
☐ Hay
☐ Grain/concentrate
☐ Other

Other - specify:

____

50 Do the goats have a constant supply of feed (i.e. ad libitum feeding)?

◯ Yes
◯ No

51 How often do you feed your goats?

◯ Once daily
◯ Twice daily
◯ Three times daily
◯ Other

Other - specify: ______________________________

52 What time(s) of the day is feed distributed? ______________________________

Milking

53 How are the does milked?

☐ By hand
☐ By machine
(If by machine, proceed to Question 54. If by hand, proceed to Question 57.)

54 Are the teat cups disinfected after milking?

○ Yes ○ No
(If yes, proceed to Question 55. If no, proceed to Question 57.)

55 How often are the teat cups disinfected? ______________________________

56 How are the teat cups disinfected? ______________________________

57. What is the milking frequency per day? Use a tick or cross to mark your answer.

	Once daily	Twice daily	Three times daily	Four or more times daily
Early lactation	○	○	○	○
Mid lactation	○	○	○	○
Late lactation	○	○	○	○

58 What time(s) of the day are the goats milked? ______________________________

59 Approximately how old is the milking parlor (or area where does are milked)? ______________________________

60 What routine practices are carried out prior to milking?

☐ None
☐ Fore-milk checked
☐ Teats cleaned of debris/dirt
☐ Teats disinfected/sanitized
☐ Gloves are worn by staff
☐ Other

Other - specify: ______________________________

61 What routine practices are carried out after milking?

- ☐ None
- ☐ Teat dip/spray
- ☐ Teat conditioner
- ☐ Other

Other - specify: __________________________

62 Do you check for mastitis?

◯ Yes ◯ No
(If yes, proceed to Question 63. If no, proceed to Question 64.)

63 What signs of mastitis do you look for?

- ☐ Swelling
- ☐ Color
- ☐ Heat
- ☐ Firmness to the touch
- ☐ Milk quality
- ☐ Other

Other - specify: __________________________

64 What is the average lactation length on your farm (in days)? __________________________

65 What is the average milk production per doe per day? __________________________

Kid Rearing

66 How are the kids reared?

- ☐ Dam-reared
- ☐ Hand-reared
- ☐ Other

(If dam-reared, proceed to Question 67. If hand-reared, proceed to Question 68.)

Other - specify: __________________________

67 How long are the kids dam-reared?

- ◯ 12 hours or less
- ◯ 13-24 hours
- ◯ 25-48 hours
- ◯ 49 hours or more

68 When are the kids removed from the dam?

- ◯ Immediately
- ◯ Within 4 hours after birth
- ◯ Between 5-12 hours after birth
- ◯ Between 25-48 hours after birth
- ◯ 49 hours or more after birth

69 Approximately how much colostrum (in ounces) is fed within the first 24 hours of life? __________________________

70 What type of colostrum do the kids receive?

- ☐ Heat-treated cow colostrum
- ☐ Raw cow colostrum
- ☐ Heat-treated goat colostrum
- ☐ Raw goat colostrum
- ☐ Powdered cow colostrum
- ☐ Powdered goat colostrum
- ☐ Other

Other - specify: __________________________________

71 Is the navel/umbilical cord disinfected shortly after birth?

◯ Yes ◯ No

72 How is the kid barn (or area where the kids are reared) kept warm?

- ☐ There is no heating
- ☐ Heating
- ☐ Insulated walls
- ☐ Heat lamps
- ☐ High pen walls to prevent drafts
- ☐ Walls on all 4 sides of the room
- ☐ Clean bedding
- ☐ Dry bedding
- ☐ Other

Other - specify: __________________________________

73 Since January 1 2019, how many goat kids have died of natural causes? __________________________________

74 Since January 1 2019, how many goat kids have been euthanized? __________________________________

75 What factors affect when the kids are weaned?

- ☐ Age
- ☐ Weight/size
- ☐ Milk availability/cost
- ☐ Solid feed availability/cost
- ☐ Other

Other - specify: __________________________________

76 Approximately how old are your kids at weaning? __________________________________

77 What is the average kid weight (in pounds) at weaning? __________________________________

78 What process is used to wean the kids?

☐ Reducing the quantity of milk over time
☐ Diluting the milk
☐ Replacing milk with solid feed
☐ Other

Other - specify: __________________________________

Disbudding

79 Are your kids disbudded?

◯ Yes ◯ No
(If yes, proceed to Question 80. If no, proceed to Question 94.)

80 Who generally performs the disbudding?

☐ Myself
☐ Staff
☐ Friends/family
☐ Paid contractor(s)
☐ Veterinarian
☐ Other

Other - specify: __________________________________

81 From whom did you learn how to disbud goat kids?

☐ No-one, I taught myself
☐ Staff
☐ Friends/family
☐ Paid contractor(s)
☐ Veterinarian
☐ Other

Other - specify: __________________________________

82 Approximately how old are the kids when they are disbudded? __________________________________

83 What disbudding method(s) is used?

☐ Cautery iron - Proceed to Question 84
☐ Caustic paste - Proceed to Question 87
☐ Liquid nitrogen
☐ Other

Other - specify: __________________________________

84 How is the iron powered?

☐ Electric
☐ Liquefied petroleum gas (LPG)
☐ Gas canister (butane)
☐ Other

Other - specify: __________________________________

85 Approximately how long is the iron held on each horn bud (total)?

◯ 1-4 seconds
◯ 5-7 seconds
◯ 8-12 seconds
◯ 13 or more seconds

86 Is the horn bud removed?

◯ Yes ◯ No

87 What is done to prevent the spread of the paste?

☐ Nothing
☐ The kids are isolated from each other
☐ The kids are kept indoors
☐ Vaseline is spread around the outside of the paste
☐ Other

Other - specify:

____

88 Is antiseptic applied to the disbudding wounds?

◯ Yes ◯ No

89 How do the kids usually behave during disbudding?

____

90 How do the kids usually behave following disbudding?

____

91 How confident are you in your own practice to disbud kids effectively without complication?

Not at all confident — Confident — Very confident

(Place a mark on the scale above)

92 How confident are you in the operator's practice to disbud kids effectively without complication?

Not at all confident — Confident — Very confident

(Place a mark on the scale above)

93 How likely are you to change your practice if there was a better method available?

Not at all likely — Likely — Very likely

(Place a mark on the scale above)

94 How much pain do you think disbudding causes?

None — Some pain — Extreme pain

(Place a mark on the scale above)

Castration

95 Are your buck kids generally castrated?

◯ Yes ◯ No
(If yes, proceed to Question 96. If no, proceed to Question 106.)

96 Why are your buck kids castrated?

☐ Used for showing
☐ Used for meat
☐ Kept as pets
☐ Other

Other - specify: __________________________________

97 Who generally performs the castration?
- ☐ Myself
- ☐ Staff
- ☐ Friends/family
- ☐ Paid contractor(s)
- ☐ Veterinarian
- ☐ Other

Other - specify: __________________________________

98 From whom did you learn how to castrate goat kids?
- ☐ No-one, I taught myself
- ☐ Staff
- ☐ Friends/family
- ☐ Paid contractor(s)
- ☐ Veterinarian
- ☐ Other

Other - specify: __________________________________

99 Approximately how old are the kids when they are castrated? __________________________________

100 Which castration method is used?
- ☐ Rubber ring/band
- ☐ Surgical
- ☐ Burdizzo
- ☐ Other

Other - specify: __________________________________

101 How do the kids usually behave during castration? __________________________________

102 How do the kids usually behave following castration? __________________________________

103 How confident are you in your own practice to castrate kids effectively without complication?

Not at all confident — Confident — Very confident

(Place a mark on the scale above)

104 How confident are you in the operator's practice to castrate kids effectively without complication?

Not at all confident — Confident — Very confident

(Place a mark on the scale above)

105 How likely are you to change your castration practice if there was a better method available?

Not at all likely | Likely | Very likely

(Place a mark on the scale above)

106 How much pain do you think castration causes?

None | Some pain | Extreme pain

(Place a mark on the scale above)

Hoof Trimming

107 Are the goat's hooves trimmed?

◯ Yes ◯ No
(If yes, proceed to Question 108. If no, proceed to Question 117.)

108 Who generally performs the hoof trimming?

- ☐ Myself
- ☐ Staff
- ☐ Friends/family
- ☐ Paid contractor(s)
- ☐ Veterinarian
- ☐ Other

Other - specify: ______________________

109 From whom did you learn how to hoof trim goats?

- ☐ No-one, I taught myself
- ☐ Staff
- ☐ Friends/family
- ☐ Paid contractor(s)
- ☐ Veterinarian
- ☐ Other

Other - specify: ______________________

110 What method is used to trim the goat's hooves?

- ☐ Grinder
- ☐ Hand-powered trimmer/shears
- ☐ Pneumatic hoof trimmer
- ☐ Blade
- ☐ Other

Other - specify: ______________________

111 What factors affect when the goat's hooves are trimmed?

- ☐ Length of hooves
- ☐ Age
- ☐ Routinely
- ☐ Once identified as lame
- ☐ Other

Other - specify: ______________________

112 Approximately how often are the goat's hooves trimmed?

____

113 Approximately how old are the goats at their first hoof trim?

____

114 How confident are you in your own practice to hoof trim goats effectively without complication?

Not at all confident — Confident — Very confident

(Place a mark on the scale above)

115 How confident are you in the operator's practice to hoof trim goats effectively without complication?

Not at all confident — Confident — Very confident

(Place a mark on the scale above)

116 How likely are you to change your practice if there was a better method available?

Not at all likely — Likely — Very likely

(Place a mark on the scale above)

Euthanasia

117 Are goats/kids euthanized on your farm?

◯ Yes ◯ No
(If yes, proceed to Question 118. If no, proceed to Question 127.)

118 Why are goats/kids euthanized?

- ☐ Fractures
- ☐ Emergency medical conditions causing severe pain
- ☐ Emaciation/debilitation from disease/injury
- ☐ Paralysis
- ☐ High cost of treatment
- ☐ Diseases with no known cure (e.g., Johne's)/recovery is too long
- ☐ Positive test for caprine arthritis encephalitis
- ☐ Unwanted buck/doe kids
- ☐ Retired/old milking doe
- ☐ Retired/old breeding buck
- ☐ Other

Other - specify:

____

119 Who generally euthanizes the goats/kids?

- ☐ Myself
- ☐ Staff
- ☐ Friends/family
- ☐ Paid contractor(s)
- ☐ Veterinarian
- ☐ Other

Other - specify:

____

120 From whom did you learn how to euthanize goats/kids?

- ☐ No-one, I taught myself
- ☐ Staff
- ☐ Friends/family
- ☐ Paid contractor(s)
- ☐ Veterinarian
- ☐ Other

Other - specify: ______________________________

121 What method(s) is used for kids?

- ☐ Blunt force trauma (e.g., with a hammer)
- ☐ Captive bolt device
- ☐ Firearm
- ☐ Veterinarian administered drug
- ☐ Other

Other - specify: ______________________________

122 What method(s) is used for adult goats?

- ☐ Blunt force trauma (e.g., with a hammer)
- ☐ Captive bolt device
- ☐ Firearm
- ☐ Veterinarian administered drug
- ☐ Other

Other - specify: ______________________________

123 How do you confirm death? ______________________________

124 How confident are you in your own practice to euthanize goats/kids effectively without complication?

Not at all confident — Confident — Very confident

(Place a mark on the scale above)

125 How confident are you in the operator's practice to euthanize animals effectively without complication?

Not at all confident — Confident — Very confident

(Place a mark on the scale above)

126 How likely are you to change your euthanasia practice if there was a better method available?

Not at all likely — Likely — Very likely

(Place a mark on the scale above)

Health

127 Are sick/injured animals housed in separate pens to the healthy animals?

◯ Yes ◯ No

128 Is pain relief used on your farm?

◯ Yes ◯ No
(If yes, proceed to Question 129. If no, proceed to Question 133.)

129 What is pain relief used for?

☐ Disbudding
☐ Castration
☐ Disease
☐ Injury
☐ Other

Other - specify:

____

130 Who administers the pain relief?

☐ Myself
☐ Staff
☐ Friends/family
☐ Paid contractor(s)
☐ Veterinarian
☐ Other

Other - specify:

____

131 Did a veterinarian provide the pain relief?

◯ Yes ◯ No

132 Did a veterinarian provide training on how to administer the pain relief?

◯ Yes ◯ No

133 What factors affect the use of pain relief on your farm?

☐ Cost
☐ Time taken to administer pain relief
☐ Use of a veterinarian
☐ Benefits for the animal
☐ Benefits for humans (i.e. ease of handling)
☐ Other

Other - specify:

____

134 How often is veterinary treatment sought?

Never — Sometimes — Always

(Place a mark on the scale above)

135 Do you feel that the veterinarian you use has adequate experience with dairy goats?

◯ Yes
◯ No

136 For what services do you usually request a veterinarian?

- ☐ Care for sick/injured animals
- ☐ Emergencies
- ☐ Herd health
- ☐ Hoof trimming
- ☐ Disbudding
- ☐ Castration
- ☐ Euthanasia
- ☐ Reproduction
- ☐ Record keeping
- ☐ Nutrition advice
- ☐ Other

Other - specify: ______________________

137 What factors affect the use of veterinarians?

- ☐ Expense
- ☐ Time
- ☐ Administration of pain relief
- ☐ Good experience with a veterinarian
- ☐ Bad experience with a veterinarian
- ☐ Local veterinary practice
- ☐ Distant veterinary practice
- ☐ The level of goat experience/knowledge
- ☐ Other

Other - specify: ______________________

138 How often are your goats treated for internal parasites (i.e. dewormed)? ______________________

139 How often are your goats treated for external parasites (e.g., flies, ticks, lice etc.)? ______________________

140 Has Caseous Lymphadenitis (CL) been diagnosed on your farm?

◯ Yes ◯ No
(If yes, proceed to Question 141. If no, proceed to Question 143.)

141 Approximately how many cases? ______________________

142 What do you do for goats testing positive? ______________________

143 Has Caprine Arthritis Encephalitis (CAE) been diagnosed on your farm?

◯ Yes ◯ No
(If yes, proceed to Question 144. If no, proceed to Question 146.)

144 Approximately how many cases? ______________________

145 What do you do for goats testing positive?

____

146 Has Johne's Disease been diagnosed on your farm?

○ Yes ○ No
(If yes, proceed to Question 147. If no, proceed to Question 149.)

147 Approximately how many cases?

____

148 What do you do for goats testing positive?

____

149 Do you observe lameness on your farm?

○ Yes ○ No
(If yes, proceed to Question 150. If no, proceed to Question 152.)

150 Do you regularly treat animals identified as being lame?

○ Yes ○ No

151 How are lame goats treated?

____

152 Is body condition assessed on your farm?

○ Yes ○ No
(If yes, proceed to Question 153. If no, proceed to Question 156.)

153 How is body condition assessed?

____

154 How often is body condition assessed?

○ Daily
○ Weekly
○ Monthly
○ Yearly
○ Other

Other - specify:

____

General

156 Is each animal permanently identified?

○ Yes ○ No
(If yes, proceed to Question 157. If no, proceed to Question 158.)

157 What method(s) are used?

☐ Ear tag
☐ Collar
☐ Tattoo
☐ Other

Other - specify: __________________________________

158 How important is cleanliness on your farm?

Not at all important — Important — Very important

(Place a mark on the scale above)

159 How often are the goat pens disinfected?

- ◯ The pens are not disinfected regularly
- ◯ Daily
- ◯ Weekly
- ◯ Monthly
- ◯ Other

Other - specify: __________________________________

160 How often are the walkways leading to and from the milking parlor disinfected?

- ◯ The walkways are not disinfected regularly
- ◯ Daily
- ◯ Weekly
- ◯ Monthly
- ◯ Other

Other - specify: __________________________________

161 How often is the milking parlor disinfected?

- ◯ The milking parlor is not disinfected regularly
- ◯ Daily
- ◯ Weekly
- ◯ Monthly
- ◯ Other

Other - specify: __________________________________

162 Are goats evaluated for fitness for travel prior to transport from your farm?

- ◯ Yes
- ◯ No

(If yes, proceed to Question 163. If no, proceed to Demographics.)

163 What do you look for in a goat that is fit to travel? __________________________________

Demographics

To which gender identity do you most identify?

- ◯ Female
- ◯ Male
- ◯ Other
- ◯ Prefer not to say

How old are you?

____
